# Clinical performance of a commercially available thymidine kinase 1 assay for diagnosis of lymphoma in 42 hospitalized horses (2017‐2020)

**DOI:** 10.1111/jvim.16239

**Published:** 2021-08-06

**Authors:** Caitlin Moore, Darko Stefanovski, Daniela Luethy

**Affiliations:** ^1^ Department of Clinical Studies University of Pennsylvania Kennett Square Pennsylvania USA; ^2^ Large Animal Clinical Sciences University of Florida College of Veterinary Medicine Gainesville Florida USA

**Keywords:** biomarker, hemolymphatic neoplasia, lymphoma, lymphosarcoma, oncology‐diagnosis

## Abstract

**Background:**

Antemortem definitive diagnosis of lymphoma in horses is often difficult. Thymidine kinase 1 (TK1) assay is a potentially useful biomarker for lymphoma in horses.

**Hypothesis/objectives:**

To report the clinical performance of a commercially available TK1 assay for diagnosis of lymphoma in horses. We hypothesized that there would be no association between serum TK1 activity and a diagnosis of lymphoma in horses.

**Animals:**

Forty‐two hospitalized horses, 14 with a definitive diagnosis of lymphoma, 4 with other neoplasia, and 24 with inflammatory disease.

**Methods:**

Retrospective medical record review, groups were compared via Kruskal‐Wallis and Mann‐Whitney tests, and logistic regression was performed.

**Results:**

Median (range) TK1 was 3 U/L (0.4‐17.7 U/L) in horses with lymphoma and 3.9 U/L (0.8‐94 U/L) in horses without lymphoma (*P* = .59). There was no significant difference in total protein between horses with and without lymphoma (6.6 g/dL [5.5‐8.3 g/dL] vs 6.6 g/dL [4.7‐10.4 g/dL]; *P* = .83). There was no significant difference in fibrinogen between horses with and without lymphoma (447 [100‐1364] mg/dL vs 433 [291‐2004] mg/dL; *P* = .47). On logistic regression, serum TK1 activity was not associated with a diagnosis of lymphoma (odds ratio, 0.97; 95% confidence interval, 0.9‐1.05, *P* = .48).

**Conclusion and Clinical Importance:**

Serum TK1 values were not predictive of lymphoma diagnosis in this cohort of horses.

**Abbreviation**
TK1: thymidine kinase 1

## 1 INTRODUCTION

Lymphoma is a common malignant neoplasia in horses, with a reported prevalence as high as 14% in some cohorts.^1^ Common clinical signs associated with lymphoma in horses are vague and variable and can include fever, lymphadenopathy, weight loss, colic, ventral or peripheral edema, cutaneous lesions, diarrhea, and/or inappetence.^2^ Antemortem diagnosis of equine lymphoma often remains a diagnostic challenge for clinicians. Because of the invasive nature of obtaining biopsies to confirm a diagnosis of lymphoma, an accurate serum biomarker would be an attractive alternative. An assay for such a biomarker, thymidine kinase 1 (TK1), has been validated for use in horses.^3^

Thymidine kinase 1 is a cytosolic enzyme involved in pyrimidine salvage during DNA synthesis.^4^ Thymidine kinase 1 is upregulated during rapid cell proliferation and serum activity of this enzyme correlates with levels of cell proliferation within neoplasms. Increased serum TK1 activity is seen associated with leakage of the enzyme from dying cells during replication and increased activity of TK1 is associated with hematologic neoplasia in humans.^5^, ^6^ Serum TK1 activity is used as a marker of lymphoma in dogs and cats.^4^, ^7^, ^8^ The use of TK1 has expanded in other species, including monitoring progression of disease and remission in dogs, cats, and humans.^9^, ^10^, ^11^, ^12^ Serum TK1 activity in horses can be transiently elevated with inflammatory conditions and is consistently elevated in horses with lymphoma.^3^ However, despite its clinical utility in other species, as well as the recent study suggesting its use in horses, our clinical impression has been that TK1 is not a clinically useful marker for lymphoma in horses. Therefore, the objective of this study was to report the results of a commercially available thymidine kinase assay in a cohort of hospitalized horses in which lymphoma was suspected or diagnosed. We hypothesized that there would be no association between serum TK1 activity and a diagnosis of lymphoma in horses.

## 2 MATERIALS AND METHODS

### 2.1 Data collection

Cases were identified by search of electronic medical records from 2017 to 2020 at the George D Widener Hospital for Large Animals at the University of Pennsylvania's New Bolton Center. Case inclusion criteria consisted of (a) horses >6 months of age with (b) serum thymidine kinase assay (VDI Labs, Simi Valley, California) and (c) cytologic or histologic diagnosis of lymphoma attempted, and/or necropsy results (where applicable). Additional information gathered from medical record review included signalment, presenting complaint, total protein, fibrinogen, diagnostics performed (biopsy, cytology, and/or necropsy), if lymphoma was identified, and any other neoplasia or underlying disease that was identified in the course of hospitalization. If lymphoma was identified, the tumor location and cell type was characterized as previously described.^2^ Horses were dichotomized as either lymphoma (based on histopathologic diagnosis) or as not lymphoma (based on a final diagnosis obtained and lack of confirmation of lymphoma on histopathologic or cytologic analysis).

### 2.2 Laboratory and clinical methods

Whole blood was collected from the jugular vein of all horses to evaluate serum TK1 activity. The serum was collected, frozen, and shipped for subsequent analysis by a commercially available TK1 assay (Veterinary Diagnostics Institute, Simi Valley, California). The LIASION TK assay (DiaSorin, Stillwater, Minnesota) is an indirect, modified 2 step, competitive chemiluminescence immunoassay for the quantitative determination of serum TK1, which has been validated for use in monitoring lymphoma in dogs as a nonradiometric assay.^8^, ^13^, ^14^ The laboratory reference range reports that a result >3.3 U/L is consistent with a diagnosis of lymphoma, while evaluation of a radioimmunoassay method in horses found a result of >2.7 U/L to be consistent with a diagnosis of lymphoma.^3^ Plasma fibrinogen concentrations were evaluated via immunoturbidometric assay by an automated analyzer (ACL Elite, Instrumentation Laboratory, Bedford, Massachusetts). Total protein was assessed by automated serum biochemistry analyzer (VITROS 350 Chemistry System, Ortho Clinical Diagnostics, Buckinghamshire, England).

### 2.3 Statistical methods

Statistical analysis was performed using standard statistical software (Stata 15.1MP, StataCorp, College Station, Texas; WinSAAM, University of Pennsylvania, Kennett Square, Pennsylvania). Data were assessed for normality using the Shapiro‐Wilk test for normality. Descriptive analyses included computation of medians and ranges of continuous variables and tabulation of categorical variables.

A Wilcoxon rank‐sum (Mann‐Whitney *U*) test was performed to test for difference between horses diagnosed with lymphoma, and those that were not. Kruskal‐Wallis test was performed to compare the groups of horses diagnosed with lymphoma, other neoplasia, or inflammatory diagnosis. Univariate logistic regression was performed to evaluate for an association of serum TK1 with a diagnosis of lymphoma. A *P*‐value of <.05 was set as the criterion for statistical significance.

## 3 RESULTS

Forty‐two horses met the inclusion criteria, with a median age of 13 years (range, 1‐28 years). There were 13 mares, 23 geldings, and 6 stallions. A diverse set of breeds were represented in this sample including: Warmbloods (12), Thoroughbreds (10), Standardbreds (6), Quarter Horses (2), American Miniature Horses (3), Arabians (2), Pony (2), Connemara (1), Norwegian Fjord (1), Morgan (1), Missouri Fox Trotter (1), and Draft cross (1). The most common presenting complaints were colic (9), weight loss (9), and fever (7). Of the 42 horses in this sample, 14 horses were diagnosed with lymphoma antemortem either via biopsy (8 horses) or at postmortem examination (6 horses). These horses were further categorized via tumor location and cell line based on previously reported description: multicentric (2), alimentary (2), cutaneous (3), mediastinal (2), central nervous system (2), ocular (1), and splenic (1).^2^ One horse in the lymphoma group had chronic lymphocytic leukemia. The remaining 28 horses were further characterized into 2 additional groups based on their final diagnosis: inflammatory (24) or other neoplasia (4). Postmortem examinations were performed and used for final diagnosis in 8/24 horses with inflammatory conditions and 4/4 horses with other neoplasia. Inflammatory conditions included: lymphoplasmacytic duodenitis and/or colitis (5), gastritis (3), gastric ulcers (2), enteritis (2), hepatitis (2), hepatic abscess and peritonitis (1), neutrophilic duodenitis (1), splenomegaly and fever of undetermined etiology (1), cellulitis (1), tenosynovitis (1), equine odontoclastic tooth resorption and hypercementosis (1), enteric salmonellosis (1), eosinophilic enteritis (1), pulmonary fibrosis (1), and endocarditis (1). Other neoplasia included: metastatic squamous cell carcinoma (2), rhabdomyosarcoma (1), and pheochromocytoma (1).

The highest value of TK1 (94 U/L) was obtained from a horse that had a pheochromocytoma (other neoplasia). Other markedly elevated values included a horse with massive splenomegaly which was accompanied by a febrile illness, though unfortunately at post mortem, a definitive diagnosis was not identified (52.4 U/L); a horse with mediastinal lymphoma (17.7 U/L); and a horse with chronic active hepatitis (10.9 U/L).

Table 1 summarizes TK1 activity and fibrinogen and total protein concentrations by final diagnosis. No significant differences were identified for any values between groups. There was no significant difference in TK1 between groups (median [range]: 3.0 U/L [0.4‐17.7 U/L] vs 3.9 U/L [0.8‐94 U/L] vs 3.9 U/L [0.9‐52.4 U/L], *P* = .87). There was no significant difference in total protein (median [range]: 6.6 g/dL [5.5‐8.3 g/dL] vs 7.5 g/dL [6.5‐7.7 g/dL] vs 6.5 g/dL [4.7‐10.4 g/dL], *P* = .45) or fibrinogen (median [range]: 447 mg/dL [100‐1364 mg/dL] vs 745 mg/dL [650‐841 mg/dL] vs 388 mg/dL [291‐2004 mg/dL], *P* = .33) between groups.

**TABLE 1:** Median (range) thymidine kinase 1 (TK1), total protein, and fibrinogen concentrations in 42 hospitalized horses with a final diagnosis of lymphoma, other neoplasia, inflammatory conditions, or noninflammatory conditions

	Lymphoma (n = 14)	Other neoplasia (n = 4)	Inflammatory (n = 24)
TK1 (U/L)	3 (0.4‐17.7)	3.9 (0.8‐94)	3.9 (0.9‐52.4)
Total protein (g/dL)	6.6 (5.5‐8.3)	7.5 (6.5‐7.7)	6.5 (4.7‐10.4)
Fibrinogen (mg/dL)	447 (100‐1364)	745 (650‐841)	388 (291‐2004)

Table 2 summarizes TK1 activity and fibrinogen and total protein concentrations for horses with and without a final diagnosis of lymphoma. There were no significant differences in TK1 between horses diagnosed with lymphoma and those without lymphoma (median [range]: 3.0 U/L [0.4‐17.7 U/L] vs 3.9 U/L [0.8‐94 U/L], *P* = .59). There were no significant differences in total protein (median [range]: 6.6 g/dL [5.5‐8.3 g/dL] vs 6.6 g/dL [4.7‐10.4 g/dL], *P* = .83) or fibrinogen (median [range]: 447 mg/dL [100‐1364 mg/dL] vs 433 mg/dL [291‐2004 mg/dL], *P* = .47) between horses with and without lymphoma.

**TABLE 2:** Median (range) thymidine kinase 1 (TK1), total protein, and fibrinogen concentrations in 42 hospitalized horses with a final diagnosis of lymphoma (n = 14) or no lymphoma (n = 28)

	Lymphoma (n = 14)	Not Lymphoma (n = 28)
TK1 (U/L)	3.0 (0.4‐17.7)	3.9 (0.8‐94)
Total Protein (g/dL)	6.6 (5.5‐8.3)	6.6 (4.7‐10.4)
Fibrinogen (mg/dL)	447 (100‐1364)	433 (291‐2004)

Table 3 summarizes TK1 activity and fibrinogen and total protein concentrations for horses with and without a final diagnosis of any neoplasia. There were no significant differences in TK1 between horses diagnosed with neoplasia and those without neoplasia (median [range]: 3.0 U/L [0.4‐94 U/L] vs 3.9 U/L [0.9‐52.4 U/L], *P* = .69). There were no significant differences in total protein (median [range]: 6.6 g/dL [5.5‐8.3 g/dL] vs 6.5 g/dL [4.7‐10.4 g/dL], *P* = .6) or fibrinogen (median [range]: 473 mg/dL [100‐1364 mg/dL] vs 388 mg/dL [291‐2004 mg/dL], *P* = 1.0) between horses with and without any neoplasia.

**TABLE 3:** Median (range) thymidine kinase 1 (TK1), total protein, and fibrinogen concentrations in 42 hospitalized horses with a final diagnosis of any neoplasia (n = 18) or no neoplasia (n = 24)

	Any neoplasia (n = 18)	No neoplasia (n = 24)
TK1 (U/L)	3 (0.4‐94)	3.9 (0.9‐52.4)
Total Protein (g/dL)	6.6 (5.5‐8.3)	6.5 (4.7‐10.4)
Fibrinogen (mg/dL)	473 (100‐1364)	388 (291‐2004)

**FIGURE 1:**
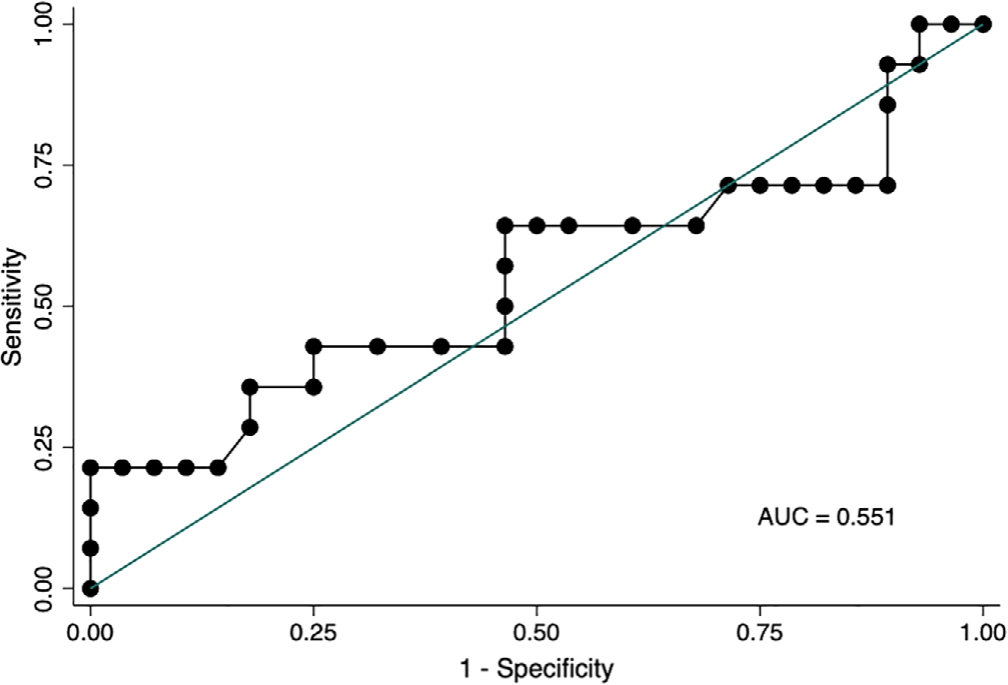
Receiver operating characteristic (ROC) curve for diagnosis of lymphoma using a commercially available serum thymidine kinase 1 assay in 42 hospitalized horses. AUC, area under the curve

Univariate logistic regression revealed no association between serum TK1 activity and a final diagnosis of lymphoma (odds ratio, 0.97; 95% confidence interval [CI], 0.9‐1.05, *P* = .48). Figure 1 shows the receiver operating characteristic curve for diagnosis of lymphoma, with an area under the curve of 0.551. Additionally, there was no association between serum TK1 activity and a final diagnosis of any neoplasia (odds ratio 1.01; 95% CI: 0.97‐1.06, *P* = .51).

## 4 DISCUSSION

In this retrospective evaluation of 42 hospitalized horses, there was no association between serum TK1 activity and a diagnosis of lymphoma. This is in contrast to the results previously reported in horses, dogs, and cats.^3^, ^10^, ^15^

The horses within our sample diagnosed with other neoplasia had a similar median serum TK1 when compared to those diagnosed with lymphoma. This could be owed to the non‐specific nature of the cytosolic enzyme which is not limited to hematopoietic cells. This is evident in hepatocellular damage in viral hepatitis, where death of hepatocytes can spill TK1 into the circulation.^16^ Two horses with hepatitis in our study had TK1 values of 7.0 U/L and 10.9 U/L, while a third horse with a hepatic abscess had a TK1 of 6.9 U/L. Given the non‐specific nature of the enzyme, it has been utilized in humans in 15 types of malignancies, both solid tumors and hematopoietic.^12^ Within our sample, no significant difference was found between horses diagnosed with any form of neoplasia and those who did not, despite a variety of tumor types being represented in the sample. In contrast, dogs with leukemia and lymphoma showed marked increases in TK1 activity, when compared to dogs with inflammatory disease, healthy controls, and those in remission.^17^ However, in another study, dogs diagnosed with B‐cell lymphoma did have higher TK1 activity than those with T‐cell lymphoma.^10^ The most common immunophenotype identified in horses is T‐cell rich large B‐cell lymphoma, as demonstrated in previous reports of equine lymphoma.^2^, ^17^, ^18^ Given this variation between immunophenotypes in canines, it could be that this specific immunophenotype in horses does not reliably cause elevations in serum TK1 activity. Therefore, another immunophenotype or neoplasm could cause more reliable elevations in TK1 activity in horses. However, it is difficult to support that assertion from our sample, as only 2 immunophenotypes were reported: 1 T‐cell rich large B‐cell and 1 B‐cell lymphoma.

Another possible explanation for the lack of association in our sample is a lack of understanding of the temporal trends of TK1 in horses with lymphoma. In contrast, serum TK1 has been utilized as a diagnostic in human oncology since the 1980s, and therefore this value has been well‐established throughout the course of disease, even preceding the diagnosis of neoplasia. In humans, TK1 elevation is associated with numerous comorbidities such as obesity, fatty liver, and pre‐cancerous lesions. Patients within this cohort also had a 3‐ to 5‐fold risk of developing cancer in the subsequent 6 years.^19^ It is therefore reasonable that horses without a histopathologic diagnosis of lymphoma that displayed elevated TK1 in our sample could represent a pre‐cancerous group. However, because our sample represents horses at a single time point, this precludes the recognition of trends over time. It also should be noted that several horses within our sample had cutaneous topography, which in dogs have been noted to have less marked TK1 activity.^10^ Some equine lymphoma subtypes might have a more indolent course, leading to inherently lower serum TK1.

Because of the retrospective nature of this study, the study was limited by small sample size, the information available within the medical record, lack of a validated assay, and a lack of follow‐up. The lack of validation of the TK1 assay used in this study presents a major limitation. The commercially available TK1 assay used in this study (chemiluminescence immunoassay) is a different method from the method previously evaluated in horses (radio enzyme immunoassay).^3^ In a previous study in dogs, the chemiluminescence assay had good correlation with the radioimmunoassay, but similar validation has not been performed in horses.^14^ Further research comparing the 2 methods in horses is warranted. The horses reflected in the study cohort likely represent selection bias, as the TK1 assay was submitted by clinicians based on a clinical indication or suspicion of lymphoma, and no normal horses were included in this study. It is possible that prior treatment could affect TK1 results, although none of the horses diagnosed with lymphoma in this study were treated before diagnosis, therefore reflecting naïve disease. Further limitations include the lack of concurrent diagnostics that could impede a definitive diagnosis in horses. Perhaps most relevantly, 11 horses in this study had only duodenal biopsies in conjunction with their serum TK1 activity. Seven of these horses were determined to have a histopathological abnormality with various cellular infiltrates: lymphoplasmacytic duodenitis (5), neutrophilic (1), and eosinophilic (1). While this is certainly a reasonable clinical diagnostic tool, it is possible that some horses were wrongly attributed to other groups (ie, inflammatory). While data do not exist regarding the sensitivity of endoscopic biopsy in diagnosis of lymphoma in horses, a recent study examined the accuracy of full thickness biopsies when compared with endoscopic biopsies in cats with alimentary tract lymphoma.^20^ A difference existed between the 2 techniques, where endoscopic biopsy was unable to definitively diagnose cats with intestinal lymphoma when compared to full thickness biopsies. However, when gastric neoplasia was the definitive diagnosis, endoscopic biopsies and full thickness biopsies were equally successful in reaching that conclusion. This speaks to the difficulty of obtaining a histopathological diagnosis in diffuse gastrointestinal lymphoma in small animal species, without resorting to more invasive biopsy techniques. This is amplified in large animal species where it has been reported that even despite exhaustive attempts to obtain an antemortem diagnosis of lymphoma, 62% could only reach the diagnosis of intestinal lymphoma at postmortem.^21^ While the availability of diagnostics has improved since the publication of that study, the clinical experience in the pursuit of a diagnosis of lymphoma in horses is still difficult for clinicians. In conclusion, this study showed no association between a diagnosis of lymphoma and serum TK1 activity in a sample of 42 hospitalized horses.

## CONFLICT OF INTEREST DECLARATION

Authors declare no conflict of interest.

## OFF‐LABEL ANTIMICROBIAL DECLARATION

Authors declare no off‐label use of antimicrobials.

## INSTITUTIONAL ANIMAL CARE AND USE COMMITTEE (IACUC) OR OTHER APPROVAL DECLARATION

Authors declare no IACUC or other approval was needed.

## HUMAN ETHICS APPROVAL DECLARATION

Authors declare human ethics approval was not needed for this study.

## References

[1] Knowles EJ, Tremaine WH, Pearson GR, Mair TS. A database survey of equine tumours in the United Kingdom. Equine Vet J. 2016;48(3):280‐284. 25594351 10.1111/evj.12421

[2] Durham AC, Pillitteri CA, San Myint M, et al. Two hundred three cases of equine lymphoma classified according to the World Health Organization (WHO) classification criteria. Vet Pathol. 2013;50(1):86‐93. 22700849 10.1177/0300985812451603

[3] Larsdotter S, Nostell K, von Euler H. Serum thymidine kinase activity in clinically healthy and diseased horses: a potential marker for lymphoma. Vet J. 2015;205(2):313‐316. 25744802 10.1016/j.tvjl.2015.01.019

[4] von Euler H, Einarsson R, Olsson U, et al. Serum thymidine kinase activity in dogs with malignant lymphoma: a potent marker for prognosis and monitoring the disease. J Vet Intern Med. 2004;18(5):696‐702. 15515587 10.1892/0891-6640(2004)18<696:stkaid>2.0.co;2

[5] Hallek M, Wanders L, Strohmeyer S, Emmerich B. Thymidine kinase: a tumor marker with prognostic value for non‐Hodgkin's lymphoma and a broad range of potential clinical applications. Ann Hematol. 1992;65(1):1‐5. 1643153 10.1007/BF01715117

[6] Hannigan BM, Barnett YA, Armstrong DB, et al. Thymidine kinases: the enzymes and their clinical usefulness. Cancer Biother. 1993;8(3):189‐197. 7804359 10.1089/cbr.1993.8.189

[7] Taylor SS, Dodkin S, Papasouliotis K, et al. Serum thymidine kinase activity in clinically healthy and diseased cats: a potential biomarker for lymphoma. J Feline Med Surg. 2013;15(2):142‐147. 23076596 10.1177/1098612X12463928 PMC10816663

[8] Von Euler HP, Rivera P, Aronsson A‐C, et al. Monitoring therapy in canine malignant lymphoma and leukemia with serum thymidine kinase 1 activity—evaluation of a new, fully automated non‐radiometric assay. Int J Oncol. 2009;34(2):505‐510. 19148486

[9] Bagegni N, Thomas S, Liu N, et al. Serum thymidine kinase 1 activity as a pharmacodynamic marker of cyclin‐dependent kinase 4/6 inhibition in patients with early‐stage breast cancer receiving neoadjuvant palbociclib. Breast Cancer Res. 2017;19(1):123. 29162134 10.1186/s13058-017-0913-7 PMC5699111

[10] Elliott JW, Cripps P, Blackwood L. Thymidine kinase assay in canine lymphoma. Vet Comp Oncol. 2013;11(1):1‐13. 22236202 10.1111/j.1476-5829.2011.00296.x

[11] Mouantri JP. Use of feline TK1 as a biomarker in disease monitoring. Swedish University of Agricultural Sciences Library; 2013, 31.

[12] Zhou J, He E, Skog S. The proliferation marker thymidine kinase 1 in clinical use. Mol Clin Oncol. 2013;1(1):18‐28. 24649117 10.3892/mco.2012.19 PMC3956229

[13] Selting KA, Sharp CR, Ringold R, Knouse J. Serum thymidine kinase 1 and C‐reactive protein as biomarkers for screening clinically healthy dogs for occult disease. Vet Comp Oncol. 2015;13(4):373‐384. 23859156 10.1111/vco.12052

[14] von Euler HP, Ohrvik AB, Eriksson SK. A non‐radiometric method for measuring serum thymidine kinase activity in malignant lymphoma in dogs. Res Vet Sci. 2006;80:17‐24. 16140350 10.1016/j.rvsc.2005.05.001

[15] Norsworthy GD, Estep JS, Hollinger C, et al. Prevalence and underlying causes of histologic abnormalities in cats suspected to have chronic small bowel disease: 300 cases (2008–2013). J Am Vet Med Assoc. 2015;247(6):629‐635. 26331421 10.2460/javma.247.6.629

[16] Tanaka K, Sishido T, Morimoto M, Inoue S, Takamura Y, Masumura M. Elevated serum thymidine kinase activity in patients with acute viral hepatitis. Gastroenterol Jpn. 1993;28(1):51‐55. 8440424 10.1007/BF02775003

[17] Miglio A, Morelli C, Gialletti R, et al. Clinical and immunophenotypic findings in 4 forms of equine lymphoma. Can Vet J. 2019;60(1):33‐40. 30651648 PMC6294024

[18] Luethy D, Frimberger AE, Bedenice D, et al. Retrospective evaluation of clinical outcome after chemotherapy for lymphoma in 15 equids (1991‐2017). J Vet Intern Med. 2019;33(2):953‐960. 30636061 10.1111/jvim.15411 PMC6430950

[19] Chen ZH, Huang SQ, Wang Y, et al. Serological thymidine kinase 1 is a biomarker for early detection of tumours—a health screening study on 35,365 people, using a sensitive chemiluminescent dot blot assay. Sensors. 2011;11(12):11064‐11080. 22247653 10.3390/s111211064 PMC3251970

[20] Evans SE, Bonczynski JJ, Broussard JD, Han E, Baer KE. Comparison of endoscopic and full‐thickness biopsy specimens for diagnosis of inflammatory bowel disease and alimentary tract lymphoma in cats. J Am Vet Med Assoc. 2006;229(9):1447‐1450. 17078807 10.2460/javma.229.9.1447

[21] Taylor S, Pusterla N, Vaughan B, et al. Intestinal neoplasia in horses. J Vet Intern Med. 2006;20:1429‐1436. 17186861 10.1892/0891-6640(2006)20[1429:inih]2.0.co;2

